# Editorial: Plant virus evolution

**DOI:** 10.3389/fpls.2026.2013082

**Published:** 2026-10-29

**Authors:** Yair Cárdenas-Conejo, Rajarshi Kumar Gaur, Youxiong Que

**Affiliations:** 1 Secretaría de Ciencia, Humanidades, Tecnología e Innovación, Gobierno de México, Ciudad de México, Mexico; 2 Centro Universitario de Investigaciones Biomédicas, Universidad de Colima, Colima, Colima, Mexico; 3 Department of Biotechnology, Deen Dayal Upadhyaya Gorakhpur University, Gorakhpur, Uttar Pradesh, India; 4 National Key Laboratory for Tropical Crop Breeding, Institute of Tropical Bioscience and Biotechnology, Sanya Research Institute, Chinese Academy of Tropical Agricultural Sciences, Sanya, China

**Keywords:** metatranscriptomics, phylogeography, plant virus, recombination, viral evolution

Plant virus evolution is shaped by processes operating across different scales. Mutation, recombination, selection, and genetic drift generate viral diversity; however, the trajectories that emerge in nature are also influenced by factors such as host availability, vector ecology, agricultural practices, landscape connectivity, and the movement of plant material. Advances in sequencing and computational approaches further increase the proportion of viral diversity that we are able to observe. The Research Topic *Plant Virus Evolution* was conceived around this multidimensional view, linking genomic change with ecological, epidemiological, and phylogeographic processes relevant to virus emergence and management.

The four contributions approach this problem from different perspectives, but together they tell a coherent story. Two studies reconstruct global virus histories, one perspective examines the ecological conditions that shape evolutionary trajectories, and one metatranscriptomic study shows how broader surveillance can reveal hidden diversity.

Li et al. examine the global evolutionary history of Wheat streak mosaic virus (WSMV), a member of the genus *Tritimovirus* in the family *Potyviridae*, by integrating complete genome sequences, coat protein sequences, and geographical records. Their analyses reveal a virus distributed across at least 26 countries on six continents and structured into distinct evolutionary lineages (**Figure 1A**). Bayesian phylogeographic reconstruction is compatible with an Old World origin, with Iran emerging as a plausible early center and Türkiye and the United States subsequently contributing to broader dissemination. However, the weak temporal signal of the available WSMV sequences limits the precision with which dates and dispersal events can be interpreted.

**Figure 1:**
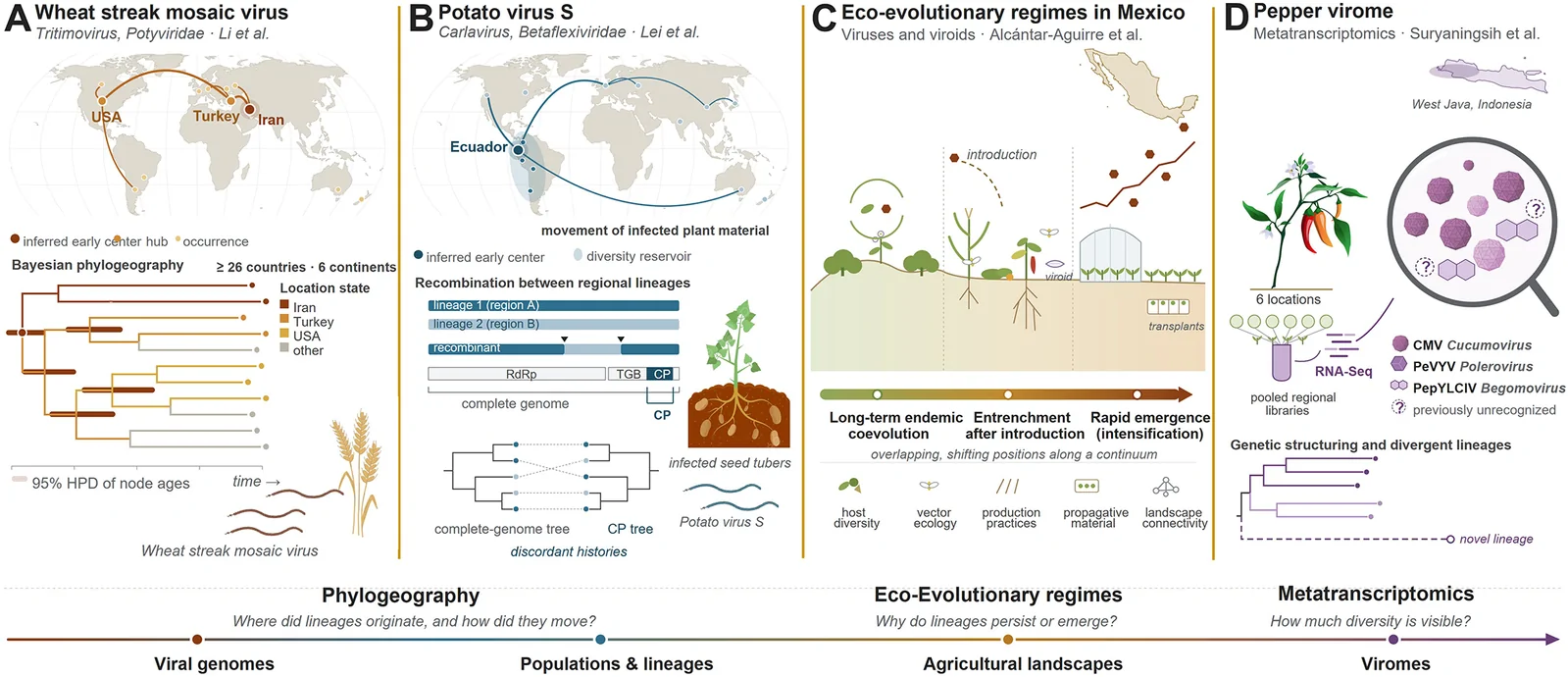
Plant virus evolution across scales, as addressed by the four contributions to this Research Topic. **(A)** The global phylogeography of the wheat streak mosaic virus, compatible with an Old World origin and Iran as a plausible early center, with Türkiye and the United States contributing to later dissemination. **(B)** Potato virus S: South America as the main reservoir of genetic diversity, recombination between regionally separated lineages, and discordance between complete-genome and coat protein phylogenies. **(C)** Mexico as a heterogeneous evolutionary landscape in which viruses and viroids occupy eco-evolutionary regimes along a continuum, shaped by host diversity, vector ecology, production practices, propagative material, and landscape connectivity. **(D)** Untargeted metatranscriptomics of pepper in West Java reveals a virome dominated by CMV, with PeVYV, PepYLCIV, and previously unrecognized sequences. Routes, tree topologies, and particle counts are schematic.

A complementary history emerges from Lei et al. for Potato virus S (PVS), a member of the genus *Carlavirus* in the family *Betaflexiviridae*. The authors’ global analysis supports South America as the principal reservoir of PVS genetic diversity and identifies Ecuador as a plausible center in its early evolutionary history, as reconstructed from current data (**Figure 1B**). Recombination between isolates from different regions illustrates how the long-distance movement of infected plant material can reconnect viral lineages that have evolved separately. Discordance between phylogenetic patterns inferred from complete genomes and from the coat protein gene also shows that evolutionary history can differ depending on the genomic window used. As with WSMV, the limited temporal signal and heterogeneous geographical sampling call for caution when translating phylogeographic inference into precise historical narratives.

These global reconstructions lead to another question: what determines whether an introduced or locally circulating virus persists, diversifies, or emerges as a major agricultural problem? Alcántar-Aguirre et al. address this by viewing Mexico as a heterogeneous evolutionary landscape rather than as a collection of independent disease outbreaks (**Figure 1C**). The authors propose three broad eco-evolutionary regimes: long-term endemic coevolution, evolutionary entrenchment following viral introduction, and rapid emergence associated with agricultural intensification. These regimes are not rigid categories, but rather positions along a continuum that may overlap or shift as ecological and agricultural conditions change. Through examples involving viruses and viroids from contrasting cropping systems, this research group shows how host diversity, vector ecology, production practices, the movement of propagative material, and landscape connectivity can create different selective environments. This perspective moves the discussion beyond the genome itself, as evolutionary potential only becomes meaningful within the ecological system in which a viral population circulates.

Suryaningsih et al. approach plant virus evolution from another direction: instead of beginning with a known virus, they ask what becomes visible when viral communities are surveyed without restricting detection to predefined targets. Using RNA-Seq-based metatranscriptomics, the authors characterize the pepper virome in six locations in West Java, Indonesia (**Figure 1D**). The profiles are dominated by Cucumber mosaic virus (CMV), a member of the genus *Cucumovirus* in the family *Bromoviridae*, while Pepper vein yellows virus, a polerovirus in the family *Solemoviridae*, and Pepper yellow leaf curl Indonesia virus, a begomovirus in the family *Geminiviridae*, occur consistently across regional libraries. Phylogenetic analyses reveal genetic structuring among some viruses, while previously unrecognized sequences expand known diversity. Because the study used pooled regional libraries, read abundance reflects viral RNA profiles rather than infection prevalence. Nevertheless, this article demonstrates how untargeted sequencing can shift the focus of plant virology from detecting individual pathogens to characterizing mixed infections, viral communities, divergent lineages, and overlooked genomic diversity.

Taken together, these contributions span a continuum from viral genomes to populations, agricultural landscapes, and entire viromes. Phylogenetics and phylogeography allow us to ask where viral lineages may have originated and how they moved. Eco-evolutionary frameworks help to explain why some lineages persist or emerge in particular hosts, vectors, and production systems. Metatranscriptomics, in turn, determines how much of the present viral diversity becomes accessible for evolutionary analysis.

This progression also exposes a challenge shared by all four contributions: our reconstruction of plant virus evolution is inseparable from the way viruses are sampled. Geographic gaps in public databases can alter inferred centers of diversity and dispersal routes; different genomic regions can produce different histories; pooled sampling can reveal community composition while limiting conclusions about individual infections; and targeted diagnostics can overlook viruses never included among the targets. Future studies will require more balanced and longitudinal sampling, the integration of host and vector information, individual-level validation where appropriate, and evolutionary models that incorporate the ecological structure of agricultural systems.

The collective message of this Research Topic is broader than the evolutionary history of any particular virus. Plant virus evolution is not only written in viral genomes. It is also written in the routes through which viruses move, in the agroecosystems in which they persist, and in the methods by which we observe them. Integrating these dimensions will be essential if evolutionary virology is to progress from reconstructing past events to anticipating future emergence and designing more durable strategies for plant virus surveillance and management.

